# Health benefits of smoke-free legislation in early and in later life

**DOI:** 10.1186/2049-3258-73-S1-K1

**Published:** 2015-09-17

**Authors:** Bianca Cox, Evelyne Martens, Benoit Nemery, Jaco Vangronsveld, Tim Nawrot

**Affiliations:** 1 UHasselt, Diepenbeek, Belgium; 2 Study Centre for Perinatal Epidemiology, Brussels, Belgium; 3 Department of Public Health and Primary Care, KU Leuven, Leuven, Belgium; 4 Hasselt University, Diepenbeek, Belgium

## Objective

In Belgium, smoke-free legislation was implemented in different phases: in public places and most workplaces in January 2006, in restaurants in January 2007, and in bars serving food in January 2010. We investigated the impact of these smoking bans on the incidence of preterm delivery and mortality of acute myocardial infarction (AMI) in Flanders. The stepwise implementation of smoke-free legislation gave us the opportunity to investigate potential successive changes in health outcomes.

## Methods

We used segmented logistic regression (preterm birth) and segmented Poisson regression (AMI mortality), adjusting for (among other things) secular trends and seasonality.

## Results

Based on 448,520 non-induced singleton live births between 2002 and 2011, we found an immediate change in the risk of preterm birth of -3.13% (95% CI -4.37 to -1.87) on 1 January 2007 (smoking ban in restaurants), and a gradual annual change of -2.65% (95% CI -5.11 to -0.13) since 1 January 2010 (smoking ban in bars serving food). These estimates correspond to a reduction of 6 preterm births per 1000 deliveries. Using data from all fatal AMIs between 2000 and 2009 (n=38,992), we observed an immediate decrease in the risk of AMI in January 2006 (smoking ban at work). The effect was largest for women younger than 60 years. Among elderly men (=60 years), we found an additional gradual effect of the smoking ban in restaurants since 1 January 2007. Our findings correspond to an estimated total of 1,715 fatal AMIs averted from 2006 to 2009.

## Conclusion

Our studies give further support that smoking bans have public health benefits not only at older age but from early life onwards. A continuation and implementation of smoke-free laws worldwide is warranted.

